# Supplementary material for: Resolving the full spectrum of human genome variation using Linked-Reads
Source: Genome Res. 2019 Apr;29(4):635–45. doi: 10.1101/gr.234443.118 (PMC6442396; doi:10.1101/gr.234443.118)
Supplement: Supplemental Material [file supp_gr.234443.118_Supplemental_longranger-2.2.zip › longranger-2.2.tar.bz2!/lr2.2-public/lib/stan/cmdstan/stan/doc/api/html/fwd_2functions_2fma_8hpp_source.html]

Stan: src/stan/agrad/fwd/functions/fma.hpp Source File


|  |  |
| --- | --- |
|  | Stan  2.5.0  probability, sampling & optimization |


- Main Page
- Related Pages
- Namespaces
- Classes
- Files

- File List
- File Members

All Classes Namespaces Files Functions Variables Typedefs Enumerations Enumerator Friends Macros Pages

- src
- stan
- agrad
- fwd
- functions

fma.hpp

Go to the documentation of this file.

1 #ifndef STAN\_\_AGRAD\_\_FWD\_\_FUNCTIONS\_\_FMA\_HPP

2 #define STAN\_\_AGRAD\_\_FWD\_\_FUNCTIONS\_\_FMA\_HPP

3

4 #include <cmath>

5 #include <stan/agrad/fwd/fvar.hpp>

6 #include <stan/meta/traits.hpp>

7

8 namespace stan {

9

10  namespace agrad {

11

59  template <typename T1, typename T2, typename T3>

60  inline

61  fvar<typename stan::return\_type<T1,T2,T3>::type>

62  fma(const fvar<T1>& x1, const fvar<T2>& x2, const fvar<T3>& x3) {

63  using ::fma;

64  return fvar<typename stan::return\_type<T1,T2,T3>::type>

65  (fma(x1.val\_, x2.val\_, x3.val\_), x1.d\_ \* x2.val\_ + x2.d\_ \* x1.val\_ + x3.d\_);

66  }

67

71  template <typename T1, typename T2, typename T3>

72  inline

73  fvar<typename stan::return\_type<T1,T2,T3>::type>

74  fma(const T1& x1, const fvar<T2>& x2, const fvar<T3>& x3) {

75  using ::fma;

76  return fvar<typename stan::return\_type<T1,T2,T3>::type>

77  (fma(x1, x2.val\_, x3.val\_), x2.d\_ \* x1 + x3.d\_);

78  }

79

83  template <typename T1, typename T2, typename T3>

84  inline

85  fvar<typename stan::return\_type<T1,T2,T3>::type>

86  fma(const fvar<T1>& x1, const T2& x2, const fvar<T3>& x3) {

87  using ::fma;

88  return fvar<typename stan::return\_type<T1,T2,T3>::type>

89  (fma(x1.val\_, x2, x3.val\_), x1.d\_ \* x2 + x3.d\_);

90  }

91

95  template <typename T1, typename T2, typename T3>

96  inline

97  fvar<typename stan::return\_type<T1,T2,T3>::type>

98  fma(const fvar<T1>& x1, const fvar<T2>& x2, const T3& x3) {

99  using ::fma;

100  return fvar<typename stan::return\_type<T1,T2,T3>::type>

101  (fma(x1.val\_, x2.val\_, x3), x1.d\_ \* x2.val\_ + x2.d\_ \* x1.val\_);

102  }

103

107  template <typename T1, typename T2, typename T3>

108  inline

109  fvar<typename stan::return\_type<T1,T2,T3>::type>

110  fma(const T1& x1, const T2& x2, const fvar<T3>& x3) {

111  using ::fma;

112  return fvar<typename stan::return\_type<T1,T2,T3>::type>

113  (fma(x1, x2, x3.val\_), x3.d\_);

114  }

115

119  template <typename T1, typename T2, typename T3>

120  inline

121  fvar<typename stan::return\_type<T1,T2,T3>::type>

122  fma(const fvar<T1>& x1, const T2& x2, const T3& x3) {

123  using ::fma;

124  return fvar<typename stan::return\_type<T1,T2,T3>::type>

125  (fma(x1.val\_, x2, x3), x1.d\_ \* x2);

126  }

127

131  template <typename T1, typename T2, typename T3>

132  inline

133  fvar<typename stan::return\_type<T1,T2,T3>::type>

134  fma(const T1& x1, const fvar<T2>& x2, const T3& x3) {

135  using ::fma;

136  return fvar<typename stan::return\_type<T1,T2,T3>::type>

137  (fma(x1, x2.val\_, x3), x2.d\_ \* x1);

138  }

139

140  }

141 }

142 #endif

stan::agrad::fma

fvar< typename stan::return\_type< T1, T2, T3 >::type > fma(const fvar< T1 > &x1, const fvar< T2 > &x2, const fvar< T3 > &x3)

The fused multiply-add operation (C99).

**Definition:** fma.hpp:62

stan::agrad::fvar

**Definition:** fvar.hpp:13

traits.hpp

fvar.hpp

stan::agrad::fma

var fma(const double &a, const stan::agrad::var &b, const stan::agrad::var &c)

The fused multiply-add function for a value and two variables (C99).

**Definition:** fma.hpp:278

stan::agrad::fvar::d\_

T d\_

**Definition:** fvar.hpp:16

stan::agrad::fvar::val\_

T val\_

**Definition:** fvar.hpp:15


---

[ Stan Home Page ]

*© 2011–2014,
Stan Development Team.*
